# Expansion of a Myeloma-associated Lesion from Orbita to the Cerebrum

**DOI:** 10.4274/tjh.2017.0283

**Published:** 2018-03-06

**Authors:** Sinan Demircioğlu, Demet Aydoğdu, Özcan Çeneli

**Affiliations:** 1 Necmettin Erbakan University Meram Faculty of Medicine, Department of Hematology, Konya, Turkey; 2 Necmettin Erbakan University Meram Faculty of Medicine, Department of Radiology, Konya, Turkey

**Keywords:** Multiple myeloma, Orbita, Cerebrum

## To the Editor,

Involvement of the central nervous system due to multiple myeloma (MM) is a very exceptional presentation with an estimated rate of 1% of all cases [1], showing a poor survival duration of 1-2 months [2,3,4]. This involvement may present in three different patterns: 1) solitary plasmacytoma, 2) multiple plasmacytomas, and 3) cerebrospinal fluid involvement with plasma cells [5].

**Figure 1:**
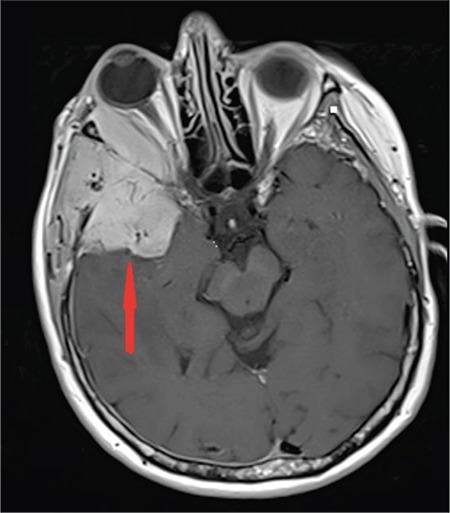
Cranial axial contrast magnetic resonance image before treatment: in the lateral aspect of the right orbit there is a mass lesion that expands and destroys the zygomatic bone and temporal lobe (red arrow). The mass lengthened in the cerebral parenchyma by invading the dura in the temporal region.

**Figure 2:**
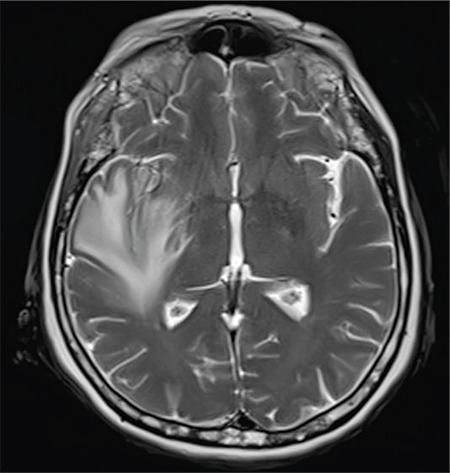
There is widespread edema (T2 axial images) around the joint due to cerebral parenchymal involvement.

**Figure 3:**
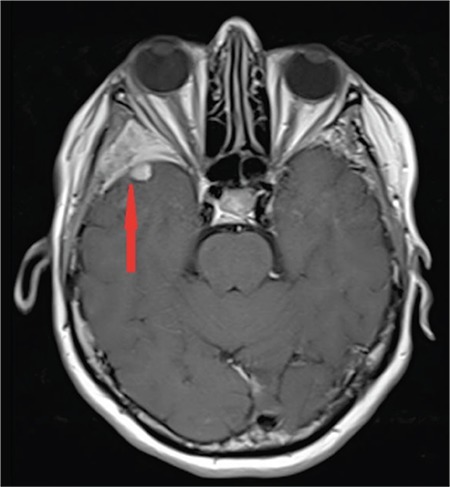
Significant regression is seen in the lesion after treatment (red arrow).

A 64-year-old female diagnosed with MM IgG kappa for 1 year was admitted with swelling and pain in the right eye. Physical examination was remarkable for proptosis. Laboratory evaluation revealed normocytic anemia, hypercalcemia, and M-protein peak in serum protein electrophoresis. Brain magnetic resonance imaging showed a retro-orbital mass of 5x6 cm in diameter extending to the right temporal region and cerebral parenchyma (Figure 1), leading to widespread edema (Figure 2). We did not evaluate the cerebrospinal fluid because it was an intracranial mass. The patient was diagnosed with recurrent MM and treated with VCD (bortezomib, cyclophosphamide, and dexamethasone). After four cycles of chemotherapy, significant clinical improvement including the regression of proptosis along with a decrease of radiological involvement was observed (Figure 3).
